# The Laron Syndrome Mouse Model Reveals a Potential Contribution of Methylglyoxal-Derived Glycative Stress to IGF-1-Driven Prostate Cancer Progression

**DOI:** 10.3390/biology15161342

**Published:** 2026-08-08

**Authors:** Dominga Manfredelli, Camilla Torcoli, Cinzia Lilli, Catia Bellucci, Vincenzo N. Talesa, Francesca Mancuso, Tiziano Baroni, Cinzia Antognelli

**Affiliations:** Department of Medicine and Surgery, University of Perugia, 06132 Perugia, Italy; dominga.manfredelli@dottorandi.unipg.it (D.M.); camilla.torcoli@collaboratori.unipg.it (C.T.); cinzia.lilli@unipg.it (C.L.); catia.bellucci@unipg.it (C.B.); vincenzo.talesa@unipg.it (V.N.T.); francesca.mancuso@unipg.it (F.M.); tiziano.baroni@unipg.it (T.B.)

**Keywords:** Laron syndrome, IGF-1, methylglyoxal, MG-H1, glycative stress, glyoxalase 1, prostate cancer, LNCaP, PC3

## Abstract

Individuals with Laron syndrome, a rare condition characterized by congenital insulin-like growth factor 1 (IGF-1) deficiency, display a remarkably low incidence of cancer, suggesting the existence of protective mechanisms linking reduced IGF-1 signaling to decreased cancer susceptibility. Consistent with this observation, IGF-1 is a recognized promoter of prostate cancer (PCa) progression, although the underlying mechanisms remain incompletely understood. Methylglyoxal (MG)-derived glycative stress, reflected by the accumulation of MG-derived hydroimidazolone 1 (MG-H1), has been implicated in PCa progression but has never been investigated in Laron syndrome. We found that liver tissues from Laron mice exhibited lower MG-H1 levels, suggesting reduced MG-derived glycative stress associated with low IGF-1 signaling. These findings prompted us to investigate whether MG-derived glycative stress contributes to IGF-1-driven PCa progression. Compared with the less aggressive LNCaP cells, PC3 cells displayed higher basal IGF-1 and MG-H1 levels, consistent with a potential association between IGF-1 and MG-derived glycative stress in PCa progression. Moreover, IGF-1 stimulation of LNCaP cells increased MG-H1 accumulation, proliferation, colony formation, invasiveness, and gene expression of matrix metalloproteinase (MMP)-1, MMP-7, MMP-9, receptor for advanced glycation end-products (RAGE), and Osteopontin (OPN), all of which were markedly attenuated by the MG scavenger aminoguanidine (AG). Collectively, these findings support a potential contribution of MG-derived glycative stress to IGF-1-driven PCa progression.

## Simple Summary

Individuals with Laron syndrome, a rare genetic condition characterized by very low levels of insulin-like growth factor 1 (IGF-1), rarely develop cancer, suggesting the existence of mechanisms that protect against cancer development. Here, we used the Laron syndrome mouse model to investigate whether reduced methylglyoxal (MG)-derived glycative stress contributes to this protective phenotype. Glycative stress results from the accumulation of advanced glycation end products (AGEs), harmful products formed when MG modifies proteins, thereby altering their function. The accumulation of these AGEs has been linked to prostate cancer (PCa) progression. We found that Laron mice had lower levels of the MG-derived glycation product MG-H1, consistent with reduced MG-derived glycative stress. Guided by this observation, we then used well-established PCa cell models with different degrees of aggressiveness and found that IGF-1 increased the levels of MG-H1, together with cell growth, colony formation, invasiveness, and the expression of molecules associated with PCa progression. Importantly, these effects were attenuated by aminoguanidine, a compound that neutralizes MG and thereby prevents MG-H1 formation. These findings suggest that MG-derived glycative stress contributes to the pro-tumorigenic effects of IGF-1 in PCa and highlight this pathway as a promising target for future studies aimed at limiting PCa progression, which remains a major clinical challenge because of its association with poor prognosis.

## 1. Introduction

Prostate cancer (PCa) is the most frequently diagnosed malignancy among men in Western countries and remains a leading cause of cancer-related mortality worldwide [1]. Although localized disease can often be successfully treated, progression toward aggressive, metastatic, and therapy-resistant stages remains a major clinical challenge and is associated with poor patient outcomes [2]. A better understanding of the molecular mechanisms driving PCa progression is therefore essential for identifying novel biomarkers and therapeutic targets to limit PCa progression.

Among the biological processes implicated in PCa progression, metabolic reprogramming has emerged as a hallmark of tumor evolution [3]. One consequence of this metabolic rewiring is the increased production of highly reactive metabolic by-products capable of modifying cellular macromolecules and influencing tumor behavior. One of these metabolites is methylglyoxal (MG), a highly reactive dicarbonyl compound mainly generated as a by-product of glycolysis [4]. In particular, MG is generated predominantly through the spontaneous degradation of the glycolytic triose phosphates glyceraldehyde-3-phosphate (G3P) and dihydroxyacetone phosphate (DHAP), two intermediates of the glycolytic pathway [4].

Under physiological conditions, MG is efficiently detoxified by the glyoxalase system, composed of glyoxalase 1 (GLO1) and glyoxalase 2 (GLO2), using glutathione (GSH) as a cofactor [5]. However, excessive MG production or impaired detoxification results in the accumulation of advanced glycation end products (AGEs), among which MG-derived hydroimidazolone 1 (MG-H1) is the predominant MG-derived adduct, accounting for more than 90% of MG-derived protein modifications [6]. Accumulation of MG-H1 is widely regarded as a hallmark of MG-derived glycative stress, which has been implicated in the pathogenesis of several diseases, including cancer [7,8]. Notably, MG-H1 has been associated with PCa progression, at least in part through activation of the receptor for advanced glycation end products (RAGE), thereby enhancing cell migration, invasion, and interactions with the tumor microenvironment [9,10,11,12,13].

Nevertheless, the upstream molecular mechanisms responsible for the induction of MG-derived glycative stress during PCa progression remain poorly understood.

Insulin-like growth factor 1 (IGF-1) is a key regulator of cell growth, survival, differentiation, and metabolism and a recognized promoter of PCa progression [14]. Elevated circulating IGF-1 levels have been associated with increased disease aggressiveness, while activation of IGF-1 signaling enhances proliferation, survival, invasion, and metastatic potential through multiple downstream pathways [14]. In addition to these well-established functions, IGF-1 stimulates glucose uptake and glycolytic metabolism through activation of the PI3K/AKT pathway [15,16,17,18], raising the possibility that sustained glycolytic activity may increase the formation of MG from glycolytic triose phosphates. However, whether this metabolic effect contributes to MG-derived glycative stress during PCa progression has not been investigated. Despite extensive evidence supporting the role of IGF-1 in PCa progression, the molecular mechanisms through which IGF-1 promotes its tumor-promoting effects remain incompletely understood. Identifying additional downstream pathways may therefore provide new opportunities for therapeutic intervention in advanced PCa.

An opportunity to address this question is provided by Laron syndrome, a rare autosomal recessive disorder caused by mutations in the growth hormone receptor (GHR) gene [19]. Because of growth hormone resistance, affected individuals exhibit congenital IGF-1 deficiency and display a remarkably low incidence of cancer despite several metabolic abnormalities [19]. This distinctive phenotype suggests the existence of protective mechanisms linking lifelong reduction of IGF-1 signaling to decreased cancer susceptibility, although the molecular basis of this protection remains largely unknown. To our knowledge, MG-derived glycative stress has never been investigated in Laron syndrome. We therefore reasoned that this rare condition could represent a valuable biological model to explore whether reduced IGF-1 signaling is associated with attenuation of MG-derived glycative stress and whether this process may contribute to the relationship between IGF-1 signaling and PCa progression.

Based on these considerations, we hypothesized that MG-derived glycative stress may contribute as a previously unrecognized downstream process to IGF-1-driven PCa progression. To test this hypothesis, we combined in vivo analyses in the Laron syndrome mouse model [19] with in vitro studies using two well-established PCa cell models differing in aggressiveness, LNCaP and PC3. Finally, using aminoguanidine as an MG scavenger [20], we pharmacologically investigated whether MG-derived glycative stress contributes to the pro-tumorigenic effects of IGF-1, including enhanced proliferation, colony formation, invasiveness, and gene expression of molecules associated with PCa progression, namely RAGE, osteopontin (OPN), and matrix metalloproteinases (MMPs).

## 2. Materials and Methods

### 2.1. Material and Treatments

RPMI-1640 culture medium, fetal bovine serum (FBS), penicillin/streptomycin, and recombinant human IGF-1 (cat. no. PHG0071) were purchased from Thermo Fisher Scientific (Milan, Italy). Recombinant human IGF-1 was reconstituted in sterile water according to the manufacturer’s instructions. Working aliquots were prepared in the presence of 0.1% bovine serum albumin (BSA) and stored at −80 °C to avoid repeated freeze–thaw cycles.

Aminoguanidine bicarbonate (AG; cat. no. 396494) and L-carnosine (Car; cat. no. C9625) were purchased from Merck (Milan, Italy). Cells were pretreated with AG at a final concentration of 1 mM for 6 h [21,22] and with Car at a final concentration of 10 mM for 48 h [12]. Recombinant human IGF-1 was then added directly to the culture medium at a final concentration of 150 ng/mL without removing AG or Car, and cells were incubated for an additional 72 h. Thus, AG or Car remained present throughout the entire period of IGF-1 stimulation. Untreated cells and cells treated with AG or Car or IGF-1 alone served as controls.

The concentration of recombinant IGF-1 (150 ng/mL) was selected on the basis of preliminary dose–response experiments, which showed a progressive increase in MG-H1 accumulation and cell proliferation across the tested concentration range (25–150 ng/mL), with the highest response observed at 150 ng/mL (Supplementary Figure S1). This concentration falls within the range of pharmacological IGF-1 concentrations used in mechanistic in vitro studies investigating IGF-1 signaling in PCa cells [23,24,25]. The aim of the present study was not to reproduce physiological circulating IGF-1 levels but rather to determine whether sustained activation of IGF-1 signaling promotes MG-derived glycative stress and the associated pro-tumorigenic phenotype. Cells were exposed continuously to 150 ng/mL IGF-1 for 72 h. IGF-1 was added once at the beginning of the experiment and was not replenished during the incubation period.

### 2.2. Laron Mouse Model and Tissue Collection

Male and female heterozygous growth hormone receptor knockout (GHR+/−) mice, originally generated in the laboratory of Prof. J. J. Kopchick (Edison Biotechnology Institute, Ohio University, Athens, OH, USA), were bred in the animal facility of the University of Perugia. Homozygous GHR−/− mice, an established experimental model of Laron syndrome characterized by congenital growth hormone resistance and markedly reduced circulating IGF-1 levels [26,27], were obtained from these breeding pairs and used in the present study. One-month-old GHR+/+ (WT) and GHR−/− mice were included in the study. Five animals were analyzed per experimental group: GHR+/+ mice (*n* = 5; 3 males and 2 females) and GHR−/− mice (*n* = 5; 3 males and 2 females). Animals were randomly selected from the available age-matched mice within each genotype. Sample allocation was based on genotype; therefore, random assignment between experimental groups was not applicable. Blinding was not performed because genotype identification was required during sample collection and processing. No animals or samples were excluded from the analysis.

All animal procedures were conducted in accordance with the Italian legislation governing the protection of animals used for scientific purposes, were approved by the Italian Ministry of Health (authorization no. 605/2019-PR, approved on 1 August 2019), and were conducted in accordance with Legislative Decree No. 26/2014 implementing Directive 2010/63/EU on the protection of animals used for scientific purposes.

Animals were euthanized under deep anesthesia, and liver samples were immediately collected.

Liver samples were collected following anesthesia induced by intraperitoneal administration of ketamine (100 mg/kg) and xylazine (10 mg/kg). Tissue samples were immediately processed for protein and RNA extraction.

For protein extraction, approximately 100 mg of tissue was homogenized in 1 mL of RIPA buffer (Santa Cruz Biotechnology, Dallas, TX, USA; cat. no. sc-24948) using an Ultra-Turrax homogenizer (IKA-Werke GmbH & Co. KG, Staufen, Germany). Homogenates were centrifuged at 2500× *g* for 10 min at 4 °C to obtain the soluble protein fraction. Total protein concentration was determined using the Bradford protein assay (Bio-Rad Laboratories, Hercules, CA, USA; cat. no. 5000006).

For RNA extraction, an additional 100 mg of tissue was homogenized in 1 mL of TRIzol™ Reagent (Thermo Fisher Scientific, Waltham, MA, USA; cat. no. 15596026), and total RNA was isolated according to the manufacturer’s instructions.

### 2.3. Cell Cultures

Human PCa cell lines LNCaP (ATCC CRL-1740, RRID: CVCL_0395) and PC3 (ATCC CRL-1435, RRID: CVCL_0035) were obtained directly from the American Type Culture Collection (ATCC, Manassas, VA, USA), authenticated by the supplier and used within 10 passages after thawing. LNCaP cells, representative of an androgen-dependent and less aggressive PCa phenotype, and PC3 cells, representative of an androgen-independent and highly aggressive phenotype, were cultured in RPMI-1640 medium (Thermo Fisher Scientific, Milan, Italy) supplemented with 10% fetal bovine serum (FBS), 100 U/mL penicillin, and 100 μg/mL streptomycin. Cells were maintained in a humidified incubator at 37 °C in an atmosphere containing 5% CO_2_ and were routinely passaged at 70–80% confluence. Cell cultures were routinely tested for mycoplasma contamination using a Mycoplasma Detection Kit (Abcam, Cambridge, UK; Cat. No. ab289834), and all cultures were confirmed to be mycoplasma-free.

### 2.4. Measurement of MG-H1 Levels

Intracellular levels of methylglyoxal-derived hydroimidazolone (MG-H1) were quantified in cell and tissue lysates using the OxiSelect™ Methylglyoxal (MG) Competitive ELISA Kit (Cell Biolabs, Inc., San Diego, CA, USA; cat. no. STA-811), according to the manufacturer’s instructions. Cell and tissue lysates were prepared using the same volume of RIPA buffer, and equal amounts of total protein were analyzed for each sample to ensure direct comparison among experimental groups. Absorbance was measured using a microplate reader at the recommended wavelength, and MG-H1 concentrations were calculated from standard curves and expressed as μg/mL.

### 2.5. Determination of Glyoxalase 1 (GLO1) Enzymatic Activity

GLO1 specific activity was determined spectrophotometrically as previously described [12,13]. Briefly, GLO1 activity was measured by monitoring the formation of S-D-lactoylglutathione from the hemithioacetal spontaneously generated by MG and GSH. The reaction was carried out in 100 mM sodium phosphate buffer (pH 6.6) containing 2 mM MG and 2 mM GSH. The increase in absorbance was monitored at 240 nm, and enzyme activity was calculated using the molar extinction coefficient of S-D-lactoylglutathione (ε = 3.3 mM^−1^ cm^−1^). GLO1 activity was normalized to total protein content, determined by the Bradford assay, and expressed as µmol/min per milligram of protein.

### 2.6. Measurement of IGF-1 Levels

Intracellular IGF-1 protein levels were determined in LNCaP and PC3 cell lysates using the Human IGF-1 ELISA Kit (Thermo Fisher Scientific, Milan, Italy; cat. no. EH250RB). The assay was performed according to the manufacturer’s instructions and adapted for the analysis of cell lysates. Cell lysates were prepared under identical experimental conditions using the same volume of RIPA buffer, and equal amounts of total protein were analyzed for each sample to ensure direct comparison between the two cell lines. Absorbance was measured at 450 nm using a microplate reader, and IGF-1 concentrations were calculated from standard calibration curves and expressed as ng/mL.

### 2.7. RNA Isolation, cDNA Synthesis, and Quantitative Real-Time PCR (qRT-PCR)

Total RNA was extracted from cultured cells and tissue samples using TRIzol™ Reagent (Thermo Fisher Scientific, Milan, Italy; cat. no. 15596026) according to the manufacturer’s instructions. RNA concentration and purity were determined spectrophotometrically by measuring the absorbance at 260 and 280 nm, while RNA integrity was verified by agarose gel electrophoresis.

First-strand cDNA was synthesized from 1 μg of total RNA using the RevertAid First Strand cDNA Synthesis Kit (Thermo Fisher Scientific, Milan, Italy; cat. no. K1622) according to the manufacturer’s instructions.

Quantitative real-time PCR (qRT-PCR) was performed on an Mx3000P Real-Time PCR System (Agilent Technologies, Milan, Italy). PCR reactions were carried out in a final volume of 20 μL containing 25 ng of cDNA, PowerTrack SYBR Green Master Mix (Thermo Fisher Scientific, Milan, Italy; cat. no. A46109) and 600 nM of each primer. Thermal cycling conditions consisted of an initial denaturation step at 95 °C for 5 min, followed by 45 amplification cycles at 95 °C for 20 s and 60 °C for 30 s. Melting curve analysis was performed for all reactions to verify amplification specificity and the absence of nonspecific PCR products.

Relative gene expression levels of IGF-1, GLO1, RAGE, OPN, MMP-1, MMP-7, and MMP-9 were normalized to the housekeeping gene β-actin and calculated using the comparative 2^−ΔΔCt^ method [28]. Primer sequences are listed in Table 1.

### 2.8. Protein Extraction and Western Blot Analysis

Thermo Scientific™ RIPA Lysis and Extraction Buffer (cat. no. 89901; Thermo Fisher Scientific, Milan, Italy) were supplemented immediately before use with Thermo Scientific™ Halt™ Protease and Phosphatase Inhibitor Cocktail (100×; cat. no. 78440; Thermo Fisher Scientific, Milan, Italy).

Western blot analysis was performed as previously described [12], with minor modifications. Equal amounts of protein (30 μg) were denatured in Laemmli sample buffer, separated by SDS-PAGE, and transferred onto nitrocellulose membranes using the iBlot™ Dry Blotting System (Thermo Fisher Scientific, Milan, Italy) according to the manufacturer’s instructions. Non-specific binding sites were blocked with Roti^®^-Block (Carl Roth, Karlsruhe, Germany) for 1 h at room temperature. Membranes were incubated overnight at 4 °C with the appropriate primary antibodies, followed by incubation for 1 h at room temperature with the corresponding horseradish peroxidase (HRP)-conjugated secondary antibodies. The antibodies used were: mouse anti-Glo1 mAb (dilution 1:1000, Santa Cruz, cat. sc-133144, DBA Italia S.r.l., Milan, Italy) and mouse anti-β-actin mAb (dilution 1:1000, Santa Cruz, cat. sc-517582, DBA Italia S.r.l., Milan, Italy). Immunoreactive bands were visualized using enhanced chemiluminescence (ECL; Amersham Pharmacia, Milan, Italy) in iBright Imaging Systems for Western blot (Thermo Fisher Scientific, Milan, Italy). β-Actin was used as the loading control.

### 2.9. Cell Proliferation Assay

Cell proliferation was assessed by direct counting of viable cells. Specifically, cells were seeded at a density of 3 × 10^5^ cells per well in 6-well plates and allowed to adhere for 24 h. Cells were then treated with AG and/or IGF-1 as indicated for 72 h. At the end of treatment, cells were harvested by trypsinization and stained with Trypan Blue, and viable cells were counted using a hemocytometer. Each experimental condition was analyzed in triplicate wells, and three independent experiments were performed. Results were expressed as the percentage of viable cells relative to untreated controls.

### 2.10. Colony Formation Assay

Following the indicated treatments, cells were seeded at low density and allowed to grow for 10 days. Colonies were then fixed with methanol, stained with crystal violet, and quantified using ImageJ software (version 1.54g, National Institutes of Health, Bethesda, MD, USA). Only colonies containing more than 50 cells were included in the analysis.

### 2.11. Cell Invasion Assay

Cell invasion was assessed using the CytoSelect 24-Well Cell Invasion Assay kit (cat. no. CBA-110, DBA Italia S.r.l., Milan, Italy) according to the manufacturer’s instructions. Following the indicated treatments, cells were seeded at a density of 5 × 10^4^ cells per insert in serum-free medium into the upper chamber, while medium containing 10% fetal bovine serum (FBS) was added to the lower chamber as a chemoattractant. Cells were allowed to invade through the matrix-coated membrane for 16 h. Non-invading cells were removed from the upper surface of the membrane, whereas invading cells were stained with crystal violet and quantified by measuring the optical density (OD) at 560 nm according to the manufacturer’s protocol.

### 2.12. Statistical Analysis

Data are presented as the mean ± standard deviation (SD) of three independent experiments. Statistical analyses were performed using GraphPad Prism version 11 (GraphPad Software, Boston, MA, USA). Comparisons between two groups were performed using Student’s *t*-test. When appropriate, comparisons among multiple groups were performed using one-way analysis of variance (ANOVA) followed by Tukey’s multiple comparisons test. Differences were considered statistically significant at *p* < 0.05.

## 3. Results

### 3.1. MG-Derived Glycative Stress Is Reduced in the Laron Syndrome Mouse Model

**Figure 1:**
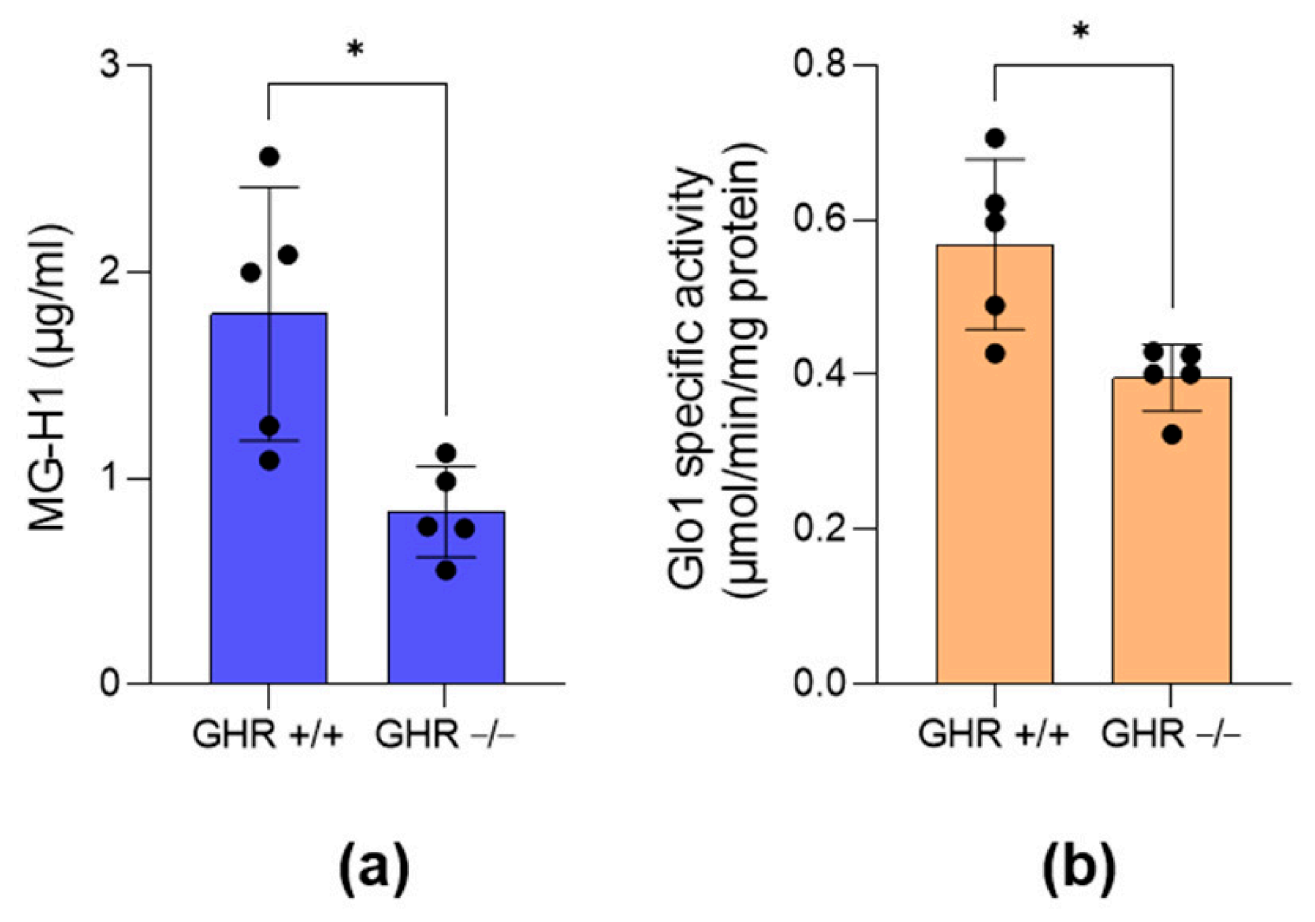
MG-H1 levels and GLO1 specific activity in the Laron syndrome mouse model. Liver homogenates from Laron (GHR−/−; *n* = 5) and control (GHR+/+; *n* = 5) mice were analyzed for (**a**) MG-H1 levels by enzyme-linked immunosorbent assay (ELISA) and (**b**) GLO1 specific activity by spectrophotometric assay. Bars represent the mean ± standard deviation (SD), and black dots represent individual mice. *p* < 0.05 versus GHR+/+ mice. * *p* < 0.05 versus GHR+/+ mice.

To investigate whether MG-derived glycative stress is altered in the Laron syndrome mouse model, MG-H1, a specific marker of MG-derived glycative stress [6], and the specific activity of glyoxalase 1 (GLO1), the rate-limiting enzyme responsible for MG detoxification [5], were evaluated in liver homogenates from Laron (GHR−/−) and control (GHR+/+) mice. Laron (GHR−/−) mice, a model of congenital IGF-1 deficiency [29], exhibited significantly lower MG-H1 levels and GLO1 specific activity than control (GHR+/+) mice (Figure 1).

These findings indicate that reduced IGF-1 signaling is associated with lower MG-derived glycative stress in the liver of Laron mice. The liver is the main organ responsible for systemic glucose metabolism and one of the principal sites of MG generation and detoxification through the glyoxalase system. To determine whether the reduction in MG-derived glycative stress observed in the liver was restricted to this organ, MG-H1 levels and GLO1 specific activity were also evaluated in dermal and skeletal muscle tissues from GHR−/− and GHR+/+ mice. Consistent with the hepatic findings, GHR−/− mice exhibited lower MG-H1 levels and lower GLO1 activity in both tissues (Supplementary Figure S2). These observations suggest that the association between congenital IGF-1 deficiency and reduced MG-derived glycative stress is not limited to the liver but is detectable across metabolically and structurally distinct tissues.

Since Laron syndrome is associated with reduced cancer susceptibility, these observations raised the possibility that attenuation of MG-derived glycative stress may represent one of the mechanisms linking low IGF-1 signaling to cancer protection. Conversely, in PCa, where both IGF-1 signaling and MG-derived glycative stress have been independently implicated in disease progression [9,10,11,12,13,14], these findings prompted us to hypothesize that the two processes may be mechanistically linked and jointly contribute to tumor progression. Based on this rationale, we next investigated whether MG-derived glycative stress contributes to the pro-tumorigenic effects of IGF-1 in PCa cells.

### 3.2. Basal IGF-1 Expression, MG-H1 Levels, and GLO1 Activity in LNCaP and PC3 Cells

**Figure 2:**
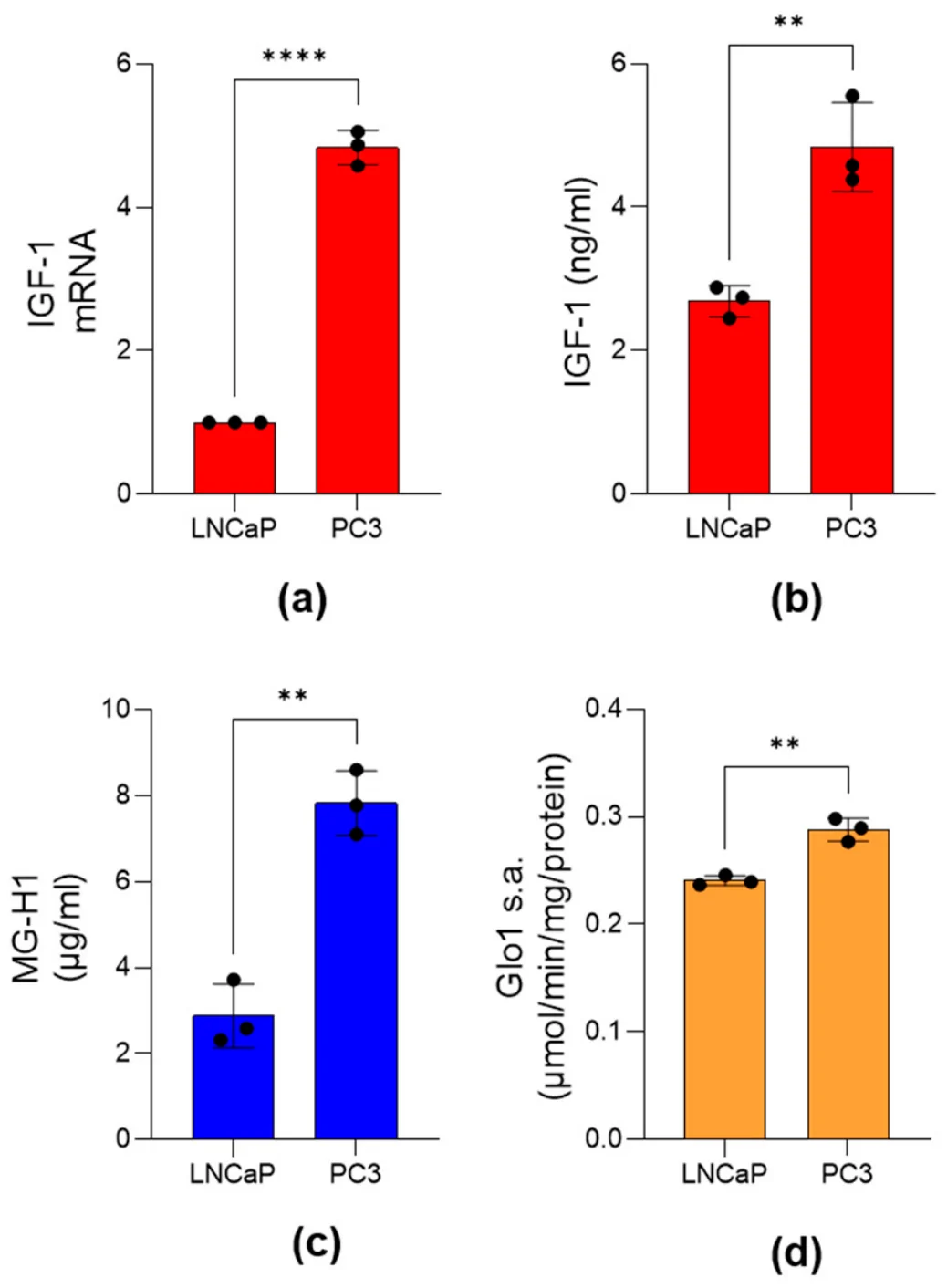
Basal IGF-1 expression is associated with increased MG-derived glycative stress in aggressive prostate cancer (PCa) cells. LNCaP and PC3 cells, representing less aggressive, androgen-dependent and more aggressive, androgen-independent human prostate cancer (PCa) cell models, respectively, were analyzed for IGF-1 expression at the (**a**) mRNA level by quantitative real-time PCR (qRT-PCR) and (**b**) intracellular protein level by enzyme-linked immunosorbent assay (ELISA), (**c**) MG-H1 levels by ELISA, and (**d**) GLO1 specific activity by spectrophotometric assay. Data are presented as the mean ± standard deviation (SD) of three independent experiments; each black dot represents one biological replicate. ** *p* < 0.01; **** *p* < 0.0001.

We first investigated whether basal IGF-1 expression is associated with MG-derived glycative stress in two established PCa cell models displaying different degrees of aggressiveness, LNCaP and PC3. To this end, IGF-1 mRNA expression, intracellular IGF-1 protein levels, MG-H1 levels, and GLO1 specific activity were evaluated. As shown in Figure 2, both IGF-1 mRNA expression (Figure 2a) and intracellular IGF-1 protein levels (Figure 2b), together with MG-H1 levels (Figure 2c), were significantly higher in the more aggressive PC3 cells than in the less aggressive LNCaP cells. Consistent with the increased MG-derived glycative stress, GLO1 specific activity was also significantly higher in PC3 cells (Figure 2d), suggesting an adaptive response to the greater MG burden.

Collectively, these findings reveal a positive association between endogenous IGF-1 expression and MG-derived glycative stress in PCa cells with different degrees of aggressiveness. While this comparison is descriptive and does not establish causality, it prompted further investigation of the relationship between sustained IGF-1 signaling and MG-derived glycative stress under controlled experimental conditions in LNCaP cells.

### 3.3. IGF-1 Induces MG-Derived Glycative Stress in LNCaP Cells

**Figure 3:**
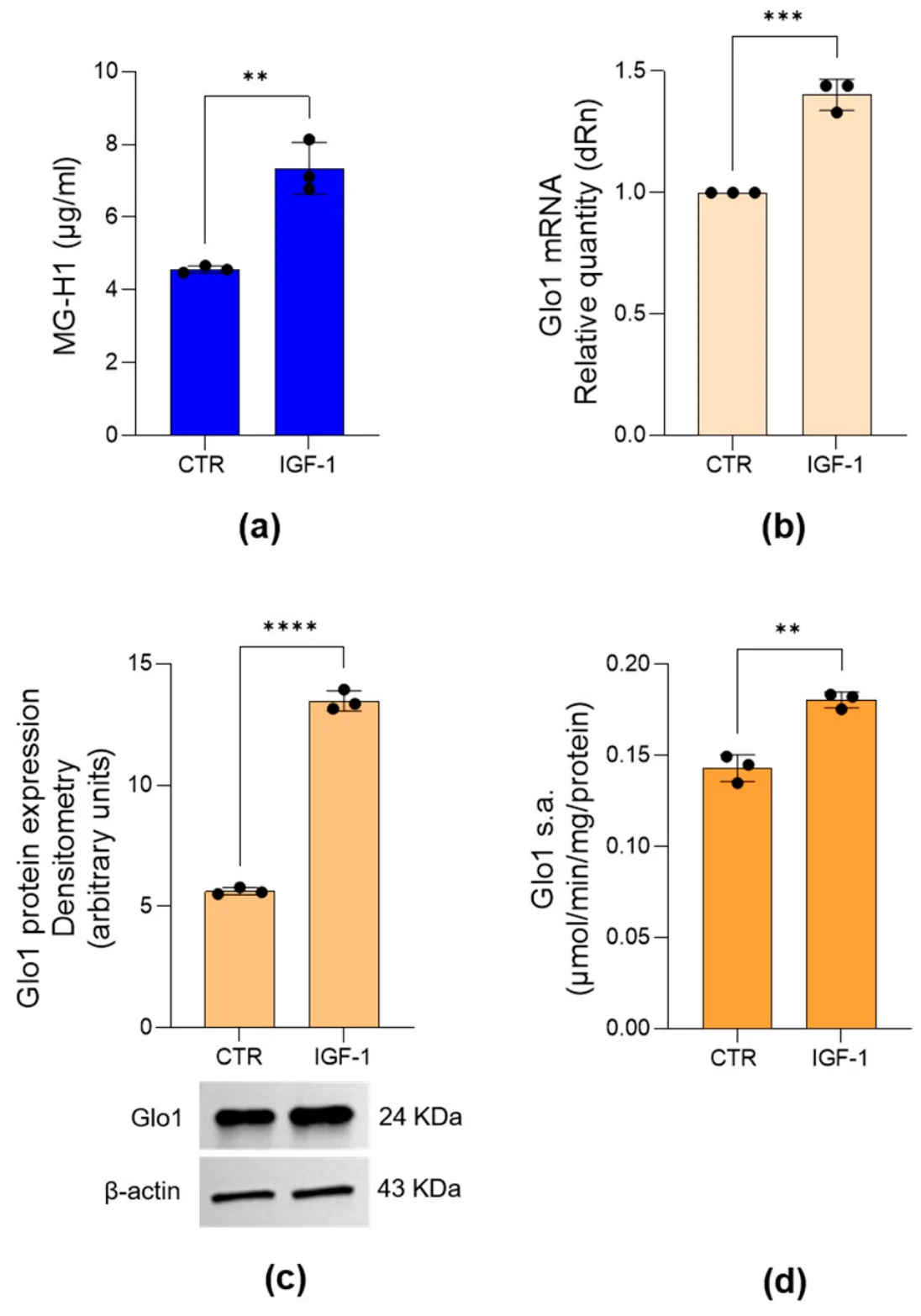
Effect of IGF-1 on MG-derived glycative stress in LNCaP cells. LNCaP cells were treated with IGF-1 (150 ng/mL) for 72 h or left untreated (control). Markers of MG-derived glycative stress were then evaluated by measuring (**a**) intracellular MG-H1 levels by enzyme-linked immunosorbent assay (ELISA), and GLO1 expression at the (**b**) mRNA level by quantitative real-time PCR (qRT-PCR), (**c**) protein level by Western blot analysis, and (**d**) functional level as GLO1 specific activity by spectrophotometric assay. β-Actin was used as the loading control for Western blot normalization. Data are presented as the mean ± standard deviation (SD) of three independent experiments; each black dot represents one biological replicate. ** *p* < 0.01; *** *p* < 0.001; **** *p* < 0.0001 versus untreated cells.

To investigate whether IGF-1 promotes MG-derived glycative stress, LNCaP cells were exposed to IGF-1 (150 ng/mL) for 72 h. As shown in Figure 3a, IGF-1 treatment significantly increased intracellular MG-H1 accumulation compared with untreated cells. In parallel, GLO1 expression was significantly upregulated at both the mRNA and protein levels, accompanied by a marked increase in GLO1 specific activity (Figure 3b–d). Collectively, these findings indicate that IGF-1 enhances MG-derived glycative stress in LNCaP cells and induces a concomitant upregulation of the glyoxalase system, likely as an adaptive response to the increased MG burden.

### 3.4. IGF-1 Enhances the Proliferative and Invasive Phenotype of LNCaP Cells

**Figure 4:**
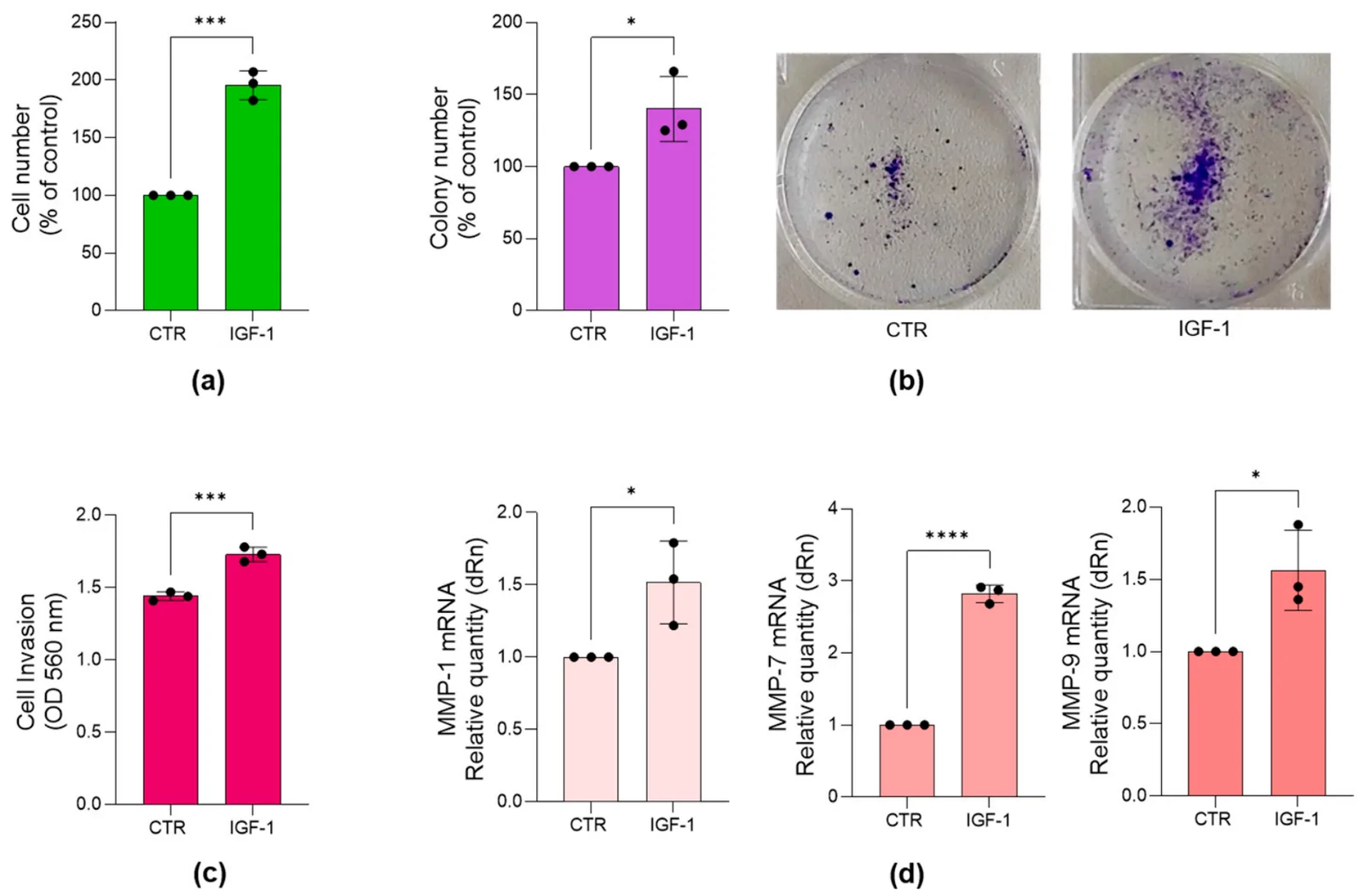
Effect of IGF-1 on the proliferative and invasive properties of LNCaP cells. LNCaP cells were treated with IGF-1 (150 ng/mL) for 72 h or left untreated (control). Cell proliferation was assessed by (**a**) cell counting and (**b**) colony formation assay. Invasive potential was evaluated by (**c**) an invasion assay and by analyzing the transcript levels of the invasion-related matrix metalloproteinases (**d**) MMP-1, MMP-7, and MMP-9. Data are presented as the mean ± standard deviation (SD) of three independent experiments; each black dot represents one biological replicate * *p* < 0.05; *** *p* < 0.001; **** *p* < 0.0001 versus untreated cells.

To investigate the biological consequences of IGF-1 stimulation, cell proliferation and invasive behavior were evaluated following exposure of LNCaP cells to IGF-1. IGF-1 treatment significantly increased cell proliferation, as shown by increased cell number and colony-forming ability compared with untreated cells (Figure 4a,b). In addition, IGF-1 markedly enhanced the invasive capacity of LNCaP cells (Figure 4c).

To further characterize this phenotype, the mRNA expression of matrix metalloproteinases (MMPs) involved in extracellular matrix remodeling and tumor invasion was evaluated. IGF-1 significantly increased the transcript levels of MMP-1, MMP-7, and MMP-9 (Figure 4d), consistent with the acquisition of a more invasive phenotype. Collectively, these findings show that IGF-1 promotes proliferative and invasive properties in LNCaP cells.

### 3.5. IGF-1 Increases RAGE and OPN mRNA Expression in LNCaP Cells

**Figure 5:**
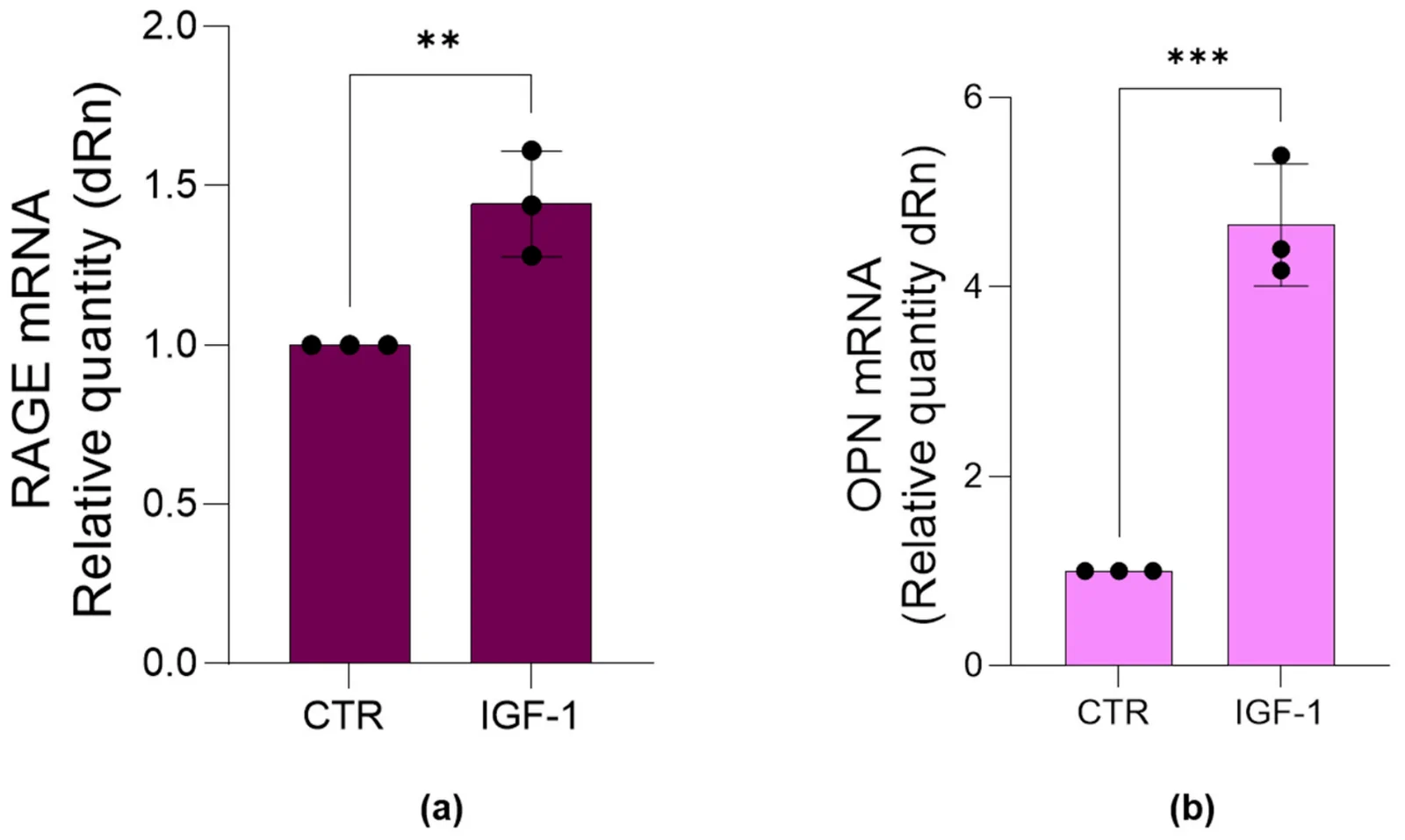
Effect of IGF-1 on RAGE and OPN mRNA expression in LNCaP cells. LNCaP cells were treated with IGF-1 (150 ng/mL) for 72 h or left untreated (control). (**a**) RAGE and (**b**) OPN mRNA expression was evaluated by quantitative real-time PCR (qRT-PCR). Data are presented as the mean ± standard deviation (SD) of three independent experiments; each black dot represents one biological replicate. ** *p* < 0.01, *** *p* < 0.001 versus untreated cells.

Since MG-H1 is a well-established ligand of the receptor for advanced glycation end products (RAGE) [30], we first investigated whether the increase in MG-derived glycative stress induced by IGF-1 was associated with changes in RAGE mRNA expression and subsequently evaluated its potential association with the expression of osteopontin (OPN). Both molecules have previously been implicated in PCa progression [13,31,32,33]. Moreover, OPN has been identified as a downstream effector of RAGE signaling in several pathological contexts [34], and the RAGE/OPN axis has been implicated in inflammatory responses associated with proliferative disorders [35]. Furthermore, an MG-H1/OPN axis has recently been proposed to contribute to the acquisition of a more aggressive phenotype in LNCaP cells [12]. As shown in Figure 5a, IGF-1 significantly increased RAGE mRNA expression in LNCaP cells. Likewise, OPN expression was markedly upregulated following IGF-1 treatment (Figure 5b).

Collectively, these findings show that IGF-1 increases the mRNA expression of both RAGE and OPN in LNCaP cells and support the hypothesis that these molecules may participate in downstream molecular events associated with PCa progression.

### 3.6. Inhibition of MG-Derived Glycative Stress by Aminoguanidine Prevents the Pro-Tumorigenic Effects of IGF-1

**Figure 6:**
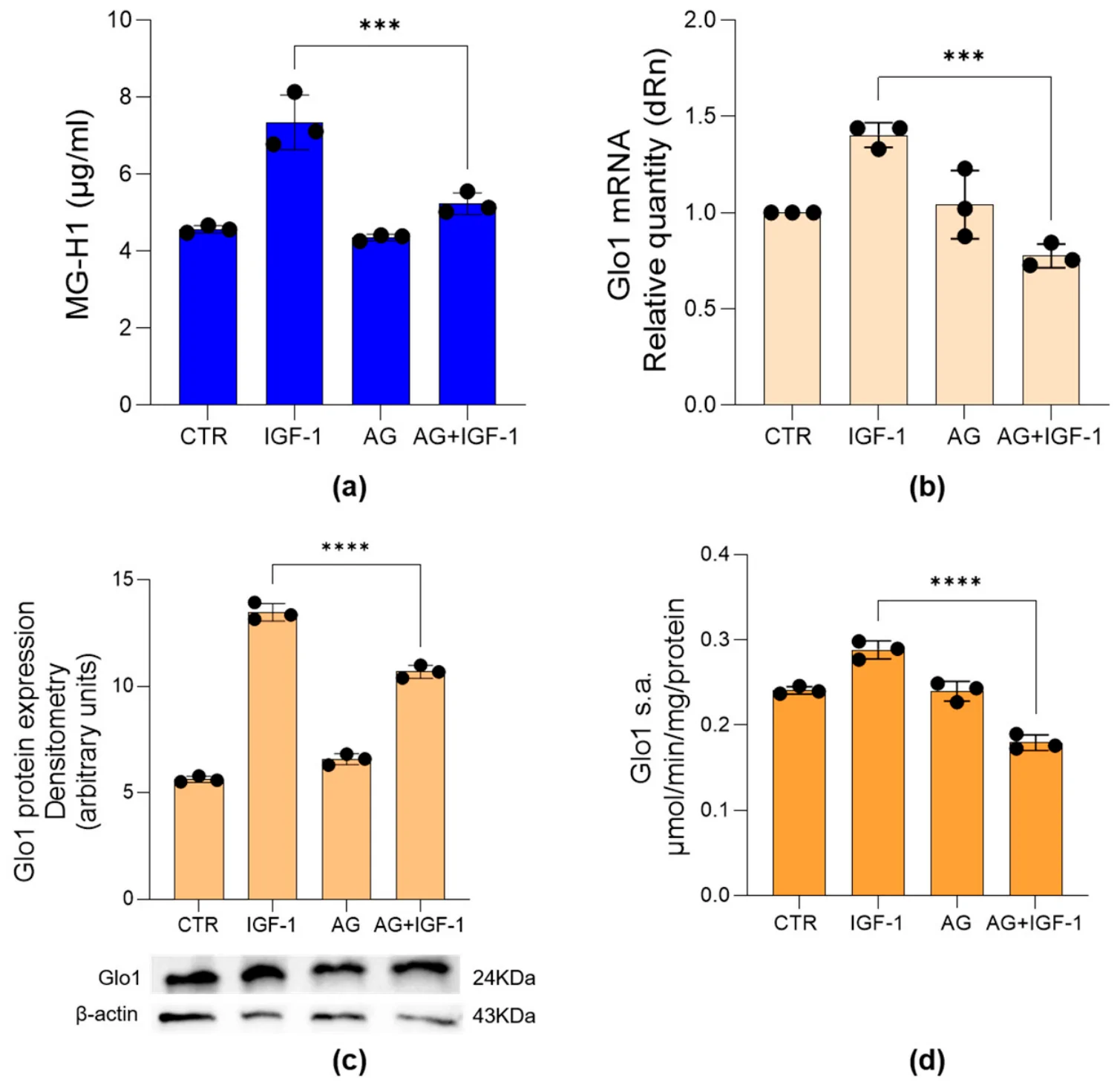
Effect of aminoguanidine on IGF-1-induced MG-derived glycative stress in LNCaP cells. LNCaP cells were pretreated with aminoguanidine (AG, 1 mM) for 6 h before exposure to IGF-1 (150 ng/mL) for 72 h. MG-derived glycative stress was evaluated by measuring (**a**) intracellular MG-H1 levels using a specific ELISA assay; GLO1 expression at the (**b**) mRNA level by qRT-PCR and (**c**) protein level by Western blot analysis; and (**d**) GLO1 enzymatic activity using a spectrophotometric assay. β-Actin was used as the loading control for normalization of Western blot data. Data are presented as the mean ± SD of three independent experiments; each black dot represents one biological replicate. *** *p* < 0.001; **** *p* < 0.0001.

To determine whether MG-derived glycative stress mediates the pro-tumorigenic effects induced by IGF-1, LNCaP cells were pretreated with aminoguanidine (AG), a well-established MG scavenger [20] that consequently limits the formation of MG-H1, before IGF-1 stimulation. As shown in Figure 6a, AG markedly reduced the intracellular accumulation of MG-H1 induced by IGF-1. Consistently, the IGF-1-mediated upregulation of GLO1 expression was also significantly attenuated by AG (Figure 6b–d), confirming the effective suppression of MG-derived glycative stress under our experimental conditions.

**Figure 7:**
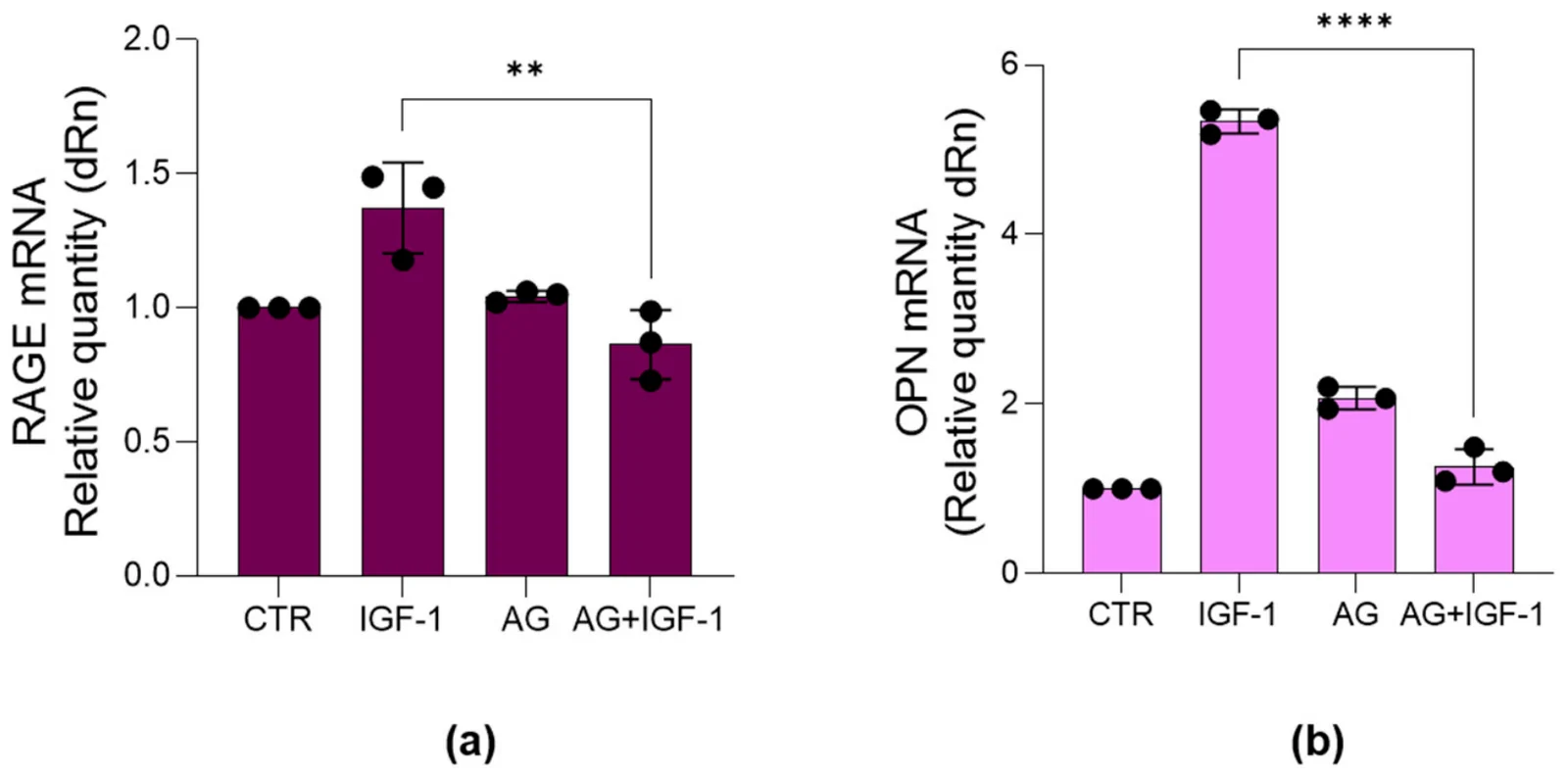
Effect of aminoguanidine on IGF-1-induced RAGE and OPN mRNA expression in LNCaP cells. LNCaP cells were pretreated with aminoguanidine (AG, 1 mM) for 6 h before exposure to IGF-1 (150 ng/mL) for 72 h. The expression of (**a**) RAGE and (**b**) osteopontin (OPN) was evaluated at the mRNA level by qRT-PCR. Data are presented as the mean ± SD of three independent experiments; each black dot represents one biological replicate. ** *p* < 0.01; **** *p* < 0.0001.

We next investigated whether inhibition of glycative stress affected the molecular changes associated with IGF-1 treatment. Pretreatment with AG significantly prevented the IGF-1-induced increase in both RAGE (Figure 7a) and OPN mRNA expression (Figure 7b), suggesting that MG-derived glycative stress contributes, at least in part, to the upregulation of these molecules.

**Figure 8:**
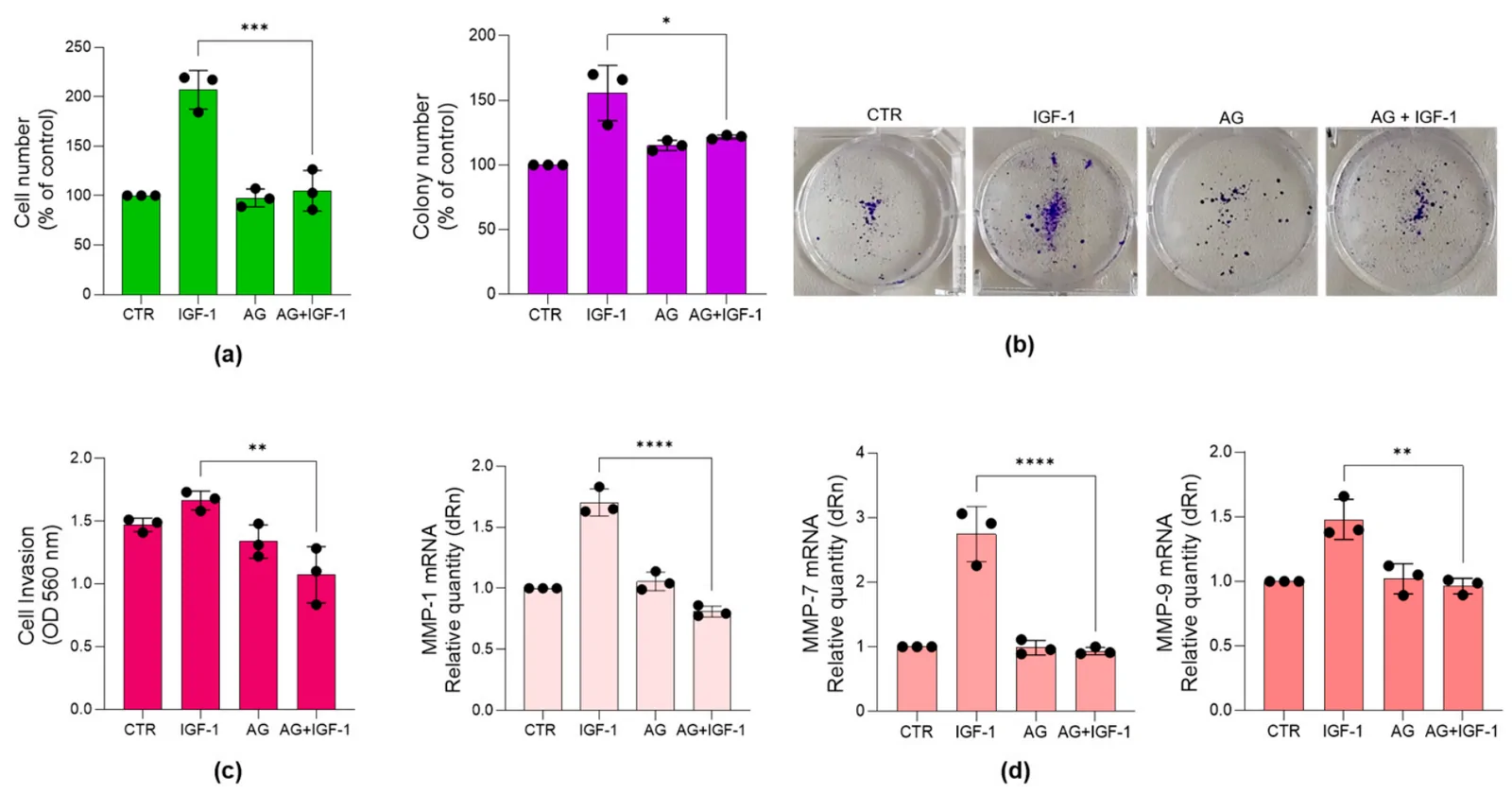
Effect of aminoguanidine on IGF-1-induced cell proliferation and invasive capacity in LNCaP cells. LNCaP cells were pretreated with aminoguanidine (AG, 1 mM) for 6 h before exposure to IGF-1 (150 ng/mL) for 72 h. Cell proliferation was evaluated by (**a**) cell counting and (**b**) colony formation assay. Invasive capacity was assessed by (**c**) a cell invasion assay and by evaluating the mRNA expression of the invasion-related metalloproteinases (**d**) MMP-1, MMP-7, and MMP-9 using qRT-PCR. Data are presented as the mean ± SD of three independent experiments; each black dot represents one biological replicate. * *p* < 0.05; ** *p* < 0.01; *** *p* < 0.001, **** *p* < 0.0001.

We then evaluated whether suppression of glycative stress influenced the biological responses elicited by IGF-1. AG significantly reduced IGF-1-induced cell proliferation (Figure 8a), clonogenic growth (Figure 8b), and invasive capacity (Figure 8c). Likewise, AG abolished the induction of the invasion-associated genes MMP-1, MMP-7, and MMP-9 observed following IGF-1 stimulation (Figure 8d).

Comparable results were obtained using the structurally unrelated MG scavenger L-carnosine [12,36,37,38] under the same treatment conditions (Supplementary Figure S3).

Collectively, these findings support the hypothesis that MG-derived glycative stress is a potential contributor to the pro-tumorigenic effects elicited by IGF-1 in LNCaP cells. Pharmacological scavenging of methylglyoxal with AG effectively suppressed MG-H1 accumulation, prevented the induction of RAGE and OPN mRNA expression, and markedly attenuated the acquisition of an aggressive phenotype. These results support a mechanistic link between IGF-1-induced glycative stress and the activation of molecular pathways associated with PCa progression.

## 4. Discussion

The present study suggests that MG-derived glycative stress, evidenced by increased MG-H1 levels, may represent a novel downstream consequence of IGF-1 signaling in PCa cells. While IGF-1 is a well-established driver of PCa progression [39,40], MG-derived glycative stress has independently been associated with tumor aggressiveness through MG-H1/RAGE-dependent mechanisms [9,10,11,12,13]. Our findings support the hypothesis that MG-derived glycative stress represents a previously unrecognized component linking IGF-1 signaling to the acquisition of a pro-tumorigenic phenotype. The observation that AG effectively prevented the IGF-1-induced increase in MG-H1 together with the associated proliferative and invasive phenotype supports the hypothesis that pharmacological targeting of MG-derived glycative stress may represent a feasible strategy for limiting IGF-1-driven PCa progression. Future studies should evaluate whether MG scavengers or other pharmacological approaches targeting this pathway may have translational potential, either alone or in combination with existing therapies [8].

Although the mechanism underlying the increase in MG-H1 following IGF-1 stimulation was not directly investigated in the present study, a plausible explanation is that enhanced IGF-1 signaling promotes metabolic conditions, thus favoring MG generation. IGF-1 is known to promote metabolic reprogramming and enhance glycolytic metabolism to sustain cell growth and proliferation [16], thereby potentially increasing the generation of MG from glycolytic intermediates. Under these conditions, MG-H1 accumulation may reflect increased MG production that exceeds the detoxification capacity of the glyoxalase system.

The simultaneous increase in MG-H1 and GLO1 is particularly intriguing. Although GLO1 detoxifies MG, its upregulation in IGF-1-treated cells likely represents an adaptive response to increased MG production rather than effective suppression of glycative stress, as also reported in highly glycolytic tumors [41]. Accordingly, the persistence of elevated MG-H1 levels despite increased GLO1 activity indicates that activation of the glyoxalase system is insufficient to counterbalance the increased MG-H1 accumulation observed following IGF-1 stimulation, thereby allowing glycative stress to contribute to the pro-tumorigenic phenotype [8].

In line with the literature, our findings indicate that IGF-1 not only stimulates tumor cell growth [23], but also promotes the acquisition of a more aggressive phenotype by enhancing invasive capacity [23] and increasing the gene expression of MMP-1, MMP-7, and MMP-9, key mediators of extracellular matrix remodeling and metastatic dissemination [42,43,44]. A previous study has demonstrated that IGF-1/IGF-1R signaling promotes PCa invasiveness through regulation of MMP-14 (MT1-MMP), a membrane-bound MMP critically involved in tumor invasion and metastasis [45]. Our findings further extend these observations by identifying MMP-1, MMP-7, and MMP-9 as additional metalloproteinases induced by IGF-1, suggesting that the pro-invasive effects of IGF-1 involve a broader extracellular matrix remodeling program than previously recognized.

MG-H1 is a well-established ligand of RAGE [30], and both RAGE and OPN have been extensively implicated in PCa progression [13,31,32,33]. Moreover, OPN has been identified as a downstream effector of RAGE signaling in several pathological contexts [34], while the RAGE/OPN axis has been associated with inflammatory responses that support proliferative disorders [35]. More recently, an MG-H1/OPN axis has also been proposed to contribute to the acquisition of a more aggressive phenotype in LNCaP cells [12]. Consistent with this evidence, we found that both RAGE and OPN gene expression was upregulated following IGF-1 stimulation and that their induction was markedly attenuated by MG scavenging. These findings support the hypothesis that MG-derived glycative stress contributes to the activation of RAGE- and OPN-associated signaling pathways downstream of IGF-1, thereby promoting tumor cell aggressiveness. However, because neither RAGE nor OPN were directly inhibited or genetically manipulated in the present study, their precise mechanistic contribution remains to be established.

The rationale of this work originated from observations obtained in experimental models of Laron syndrome, a rare disorder characterized by congenital IGF-1 deficiency and remarkable protection against cancer development [19,46,47,48]. In these models, reduced MG-H1 levels together with lower GLO1 activity suggested an attenuation of MG-derived glycative stress in the setting of chronic IGF-1 deficiency. This observation led us to hypothesize that IGF-1 may positively regulate MG-derived glycative stress pathways and that this mechanism could contribute to tumor progression. Our findings in PCa cells support this hypothesis by identifying MG-derived glycative stress as a potential downstream consequence of IGF-1 signaling associated with a pro-tumorigenic phenotype. To the best of our knowledge, this is the first study to propose that attenuation of MG-derived glycative stress may contribute to the cancer-protective phenotype associated with congenital IGF-1 deficiency.

Although the reduced MG-derived glycative stress was observed in an experimental model of Laron syndrome and therefore cannot be directly extrapolated to humans, these findings raise the intriguing possibility that attenuation of MG-H1 accumulation may play a role in cancer protection associated with IGF-1 deficiency. This hypothesis warrants further investigation in appropriate experimental and clinical settings.

More broadly, the present study illustrates the value of rare diseases as biological models for generating mechanistic hypotheses relevant to common non-communicable diseases [49]. By revealing a previously unrecognized link between IGF-1 signaling and MG-derived glycative stress, the Laron syndrome model provided the conceptual framework that led to the identification of MG-H1 as a potential contributor to PCa progression. Overall, these findings further support the concept that the metabolic reprogramming associated with IGF-1 signaling extends beyond enhanced glycolysis to include changes consistent with increased MG-derived glycative stress, thereby identifying this pathway as a functionally relevant contributor to PCa progression.

### Study Limitations

The present study has some limitations that should be considered when interpreting the findings.

The first one relates to the IGF-1 treatment conditions used in this study. Although 150 ng/mL represents a pharmacological concentration, it was selected to achieve sustained activation of IGF-1 signaling under controlled experimental conditions. Accordingly, our findings should be interpreted as reflecting the consequences of sustained IGF-1 pathway activation under controlled experimental conditions rather than physiological IGF-1 exposure. Future studies should determine whether comparable effects can be achieved under conditions of lower IGF-1 stimulation or more physiologically relevant experimental systems.

Second, the contribution of MG-derived glycative stress was investigated using two structurally unrelated MG scavengers, aminoguanidine (AG) and L-carnosine (Car). Both compounds produced comparable inhibitory effects on the IGF-1-induced phenotype, supporting the involvement of MG-derived glycative stress rather than an effect specific to a single scavenger. Nevertheless, both agents may exert biological activities beyond MG scavenging. Therefore, although these findings strengthen the evidence that MG-derived glycative stress contributes to the pro-tumorigenic effects of sustained IGF-1 signaling, they should not be interpreted as demonstrating that this pathway alone fully accounts for the biological effects elicited by IGF-1. Future studies employing complementary pharmacological approaches, direct modulation of intracellular MG levels, and genetic manipulation of GLO1 will be required to further define the mechanistic role of this pathway.

Third, MG-H1 levels and GLO1 activity were not evaluated in prostate tissue from GHR−/− mice. Although comparable changes were observed in liver, dermis and skeletal muscle, indicating that attenuation of MG-derived glycative stress is a systemic feature associated with chronic IGF-1 deficiency rather than an organ-specific phenomenon, these findings do not demonstrate that the IGF-1–MG-derived glycative stress axis operates similarly in the prostate in vivo. Moreover, the present study does not establish that the reduced cancer susceptibility associated with congenital IGF-1 deficiency is causally mediated by attenuation of MG-derived glycative stress. Accordingly, our observations should be regarded as providing a biological rationale and a framework for hypothesis generation rather than direct mechanistic evidence. Future studies examining prostate tissue and appropriate in vivo models of PCa will be required to determine whether reduced MG-derived glycative stress contributes to the cancer-protective phenotype associated with Laron syndrome. Consequently, the findings obtained in liver, dermis and skeletal muscle tissue should not be directly extrapolated to prostate tissue or interpreted as evidence that attenuation of MG-derived glycative stress explains the reduced cancer susceptibility associated with congenital IGF-1 deficiency. Future studies examining prostate tissue and appropriate in vivo models of PCa will be required to determine whether reduced MG-derived glycative stress contributes to the cancer-protective phenotype associated with Laron syndrome.

Fourth, several mechanistic aspects remain to be established. RAGE, OPN, and MMP expression levels were evaluated exclusively at the mRNA level. Although the observed transcriptional changes are consistent with the phenotypic effects induced by IGF-1 and their attenuation following MG scavenging, the corresponding protein expression and functional activity were not assessed. Likewise, intracellular MG levels and glycolytic flux were not directly quantified. Furthermore, although pharmacological MG scavenging supported the involvement of MG-derived glycative stress, complementary genetic approaches targeting GLO1, RAGE, or OPN were not performed. Therefore, the mechanistic interpretation of our findings should be considered preliminary and will require further validation using complementary biochemical, metabolic, and genetic approaches.

## 5. Conclusions

Our findings identify MG-derived glycative stress as a potential downstream consequence of IGF-1 signaling that contributes to the acquisition of a pro-tumorigenic phenotype in PCa cells. By demonstrating that pharmacological scavenging of MG attenuates the pro-tumorigenic effects of IGF-1, this study provides a rationale for further investigating whether targeting MG-derived glycative stress may represent a pathway warranting further investigation in appropriate preclinical models.

Beyond its implications for PCa biology, this work highlights the value of rare diseases as biological models for generating mechanistic hypotheses relevant to common non-communicable diseases. The observations obtained in the Laron syndrome model served as the starting point for investigating a previously unexplored relationship between IGF-1 signaling and MG-derived glycative stress in PCa. These findings illustrate how research on rare disorders can generate biologically relevant hypotheses and identify novel molecular pathways with potential translational implications extending beyond the rare disease itself.

The present findings support a previously unexplored association between sustained IGF-1 stimulation, MG-H1 accumulation, and the acquisition of proliferative and invasive properties in LNCaP PCa cells. The attenuation of these responses by aminoguanidine is consistent with a potential contribution of MG-derived glycative stress to the biological effects of IGF-1 but does not establish MG-H1 as their exclusive or direct mediator.

The reduced hepatic MG-H1 levels observed in GHR−/− mice provided the hypothesis-generating basis for the in vitro studies but cannot be directly extrapolated to prostate tissue or to the cancer-protective phenotype associated with Laron syndrome. Moreover, intracellular MG production, glycolytic flux, and the functional involvement of GLO1, RAGE, and OPN were not directly assessed. Further studies using independent genetic and pharmacological approaches, together with appropriate in vivo PCa models, will therefore be required to validate the proposed pathway and determine its biological and translational relevance.

## Figures and Tables

**Table 1:** Primer sequences used for quantitative real-time PCR (qRT-PCR).

Gene	Forward Primer (5′→3′)	Reverse Primer (5′→3′)
*IGF-1*	GCTCTTCAGTTCGTGTGTGGA	GCCTCCTTAGATCACAGCTCC
*GLO1*	CTCTCCAGAAAAGCTACACTTTGAG	CGAGGGTCTGAATTGCCATTG
*RAGE*	TGAAGGAACAGACCAGGAGACAC	GCACAGGCTCCCAGACAC
*OPN*	GCCGAGGTGATAGTGTGGTT	TGAGGTGATGTCCTCGTCTG
*MMP-1*	TTGGGCTGAAAGTGACTGGGAAAC	GGCATGGTCCACATCTGCTCTTG
*MMP-7*	GCATTTCAGGAAAGTTGTATGGG	CATCCGTCCAGCGTTCATCC
*MMP-9*	GGCAAGGGCGTCGTGGTTCC	CGGTCGTCGGTGTCGTAGTTGG
*β-actin*	CACTCTTCCAGCCTTCCTTCC	ACAGCACTGTGTTGGCGTAC

## Data Availability

The original contributions presented in this study are included in the article/Supplementary Material. Further inquiries can be directed to the corresponding author.

## Supplementary Materials

The following supporting information can be downloaded at: https://www.mdpi.com/article/10.3390/biology15161342/s1, Figure S1: Preliminary dose–response analysis used to select the IGF-1 concentration employed in the study; Figure S2: MG-H1 levels and GLO1 specific activity in dermal and skeletal muscle tissues from the Laron syndrome mouse model; Figure S3: L-Carnosine reproduces the inhibitory effects of aminoguanidine on the IGF-1-induced pro-tumorigenic phenotype in LNCaP cells; Figure S4: Uncropped western blot images and original colony formation assay plates.

## Abbreviations

The following abbreviations are used in this manuscript:

IGF-1	Insulin-like growth factor 1
MG	Methylglyoxal
MG-H1	MG-derived hydroimidazolone 1
PCa	Prostate cancer
MMP	Matrix metalloproteinase
RAGE	Receptor for advanced glycation end-products
OPN	Osteopontin
AG	Aminoguanidine
GLO1	Glyoxalase 1
GLO2	Glyoxalase 2
GSH	Glutathione
GHR	Growth hormone receptor
