# Supplementary material for: The Laron Syndrome Mouse Model Reveals a Potential Contribution of Methylglyoxal-Derived Glycative Stress to IGF-1-Driven Prostate Cancer Progression
Source: Biology (Basel). 2026 Aug 8;15(16):1342. doi: 10.3390/biology15161342 (PMC13509749; doi:10.3390/biology15161342)

Supplements: The Laron Syndrome Mouse Model Reveals a Potential Contribution of Methylglyoxal-Derived Glycative Stress to IGF-1-Driven Prostate Cancer Progression

Dominga Manfredelli, Camilla Torcoli, Cinzia Lilli, Catia Bellucci, Vincenzo N. Talesa, Francesca Mancuso, Tiziano Baroni, Cinzia Antognelli

Figure S1. Preliminary dose–response analysis used to select the IGF-1 concentration employed in the study. LNCaP cells were exposed to increasing concentrations of IGF-1 (25, 50, 100, and 150 ng/mL) for 72 h. MG-H1 accumulation and cell proliferation increased in a concentration-dependent manner, with the highest response observed at 150 ng/mL among the concentrations tested. Data are presented as mean ± SD from three independent experiments. Statistical analysis was performed by one-way ANOVA followed by Dunnett's multiple comparisons test, with each treatment compared with the untreated control. *$p < 0.05$, **$p < 0.01$, ***$p < 0.001$, ****$p < 0.0001$ vs. untreated control. These experiments were performed before initiation of the present study to define the experimental conditions used throughout the manuscript.

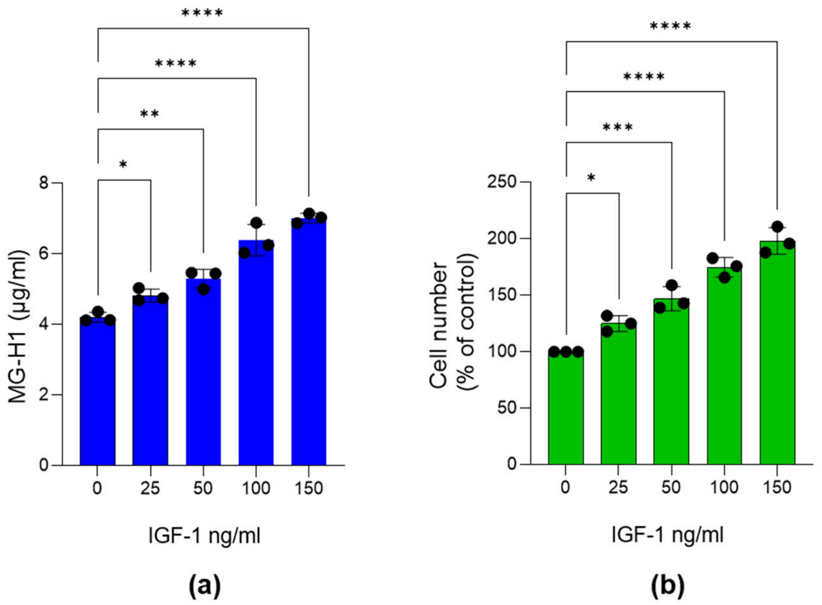

Figure S2. MG-H1 levels and GLO1 specific activity in dermal and skeletal muscle homogenates from the Laron syndrome mouse model. Dermal and skeletal muscle homogenates from Laron (GHR−/−; n = 5) and control (GHR+/+; n = 5) mice were analyzed for **(a)** MG-H1 levels by enzyme-linked immunosorbent assay (ELISA) and **(b)** GLO1 specific activity by spectrophotometric assay. Data are presented as the mean ± standard deviation (SD). **$p < 0.01$ and **** $p < 0.0001$ versus GHR+/+ mice.

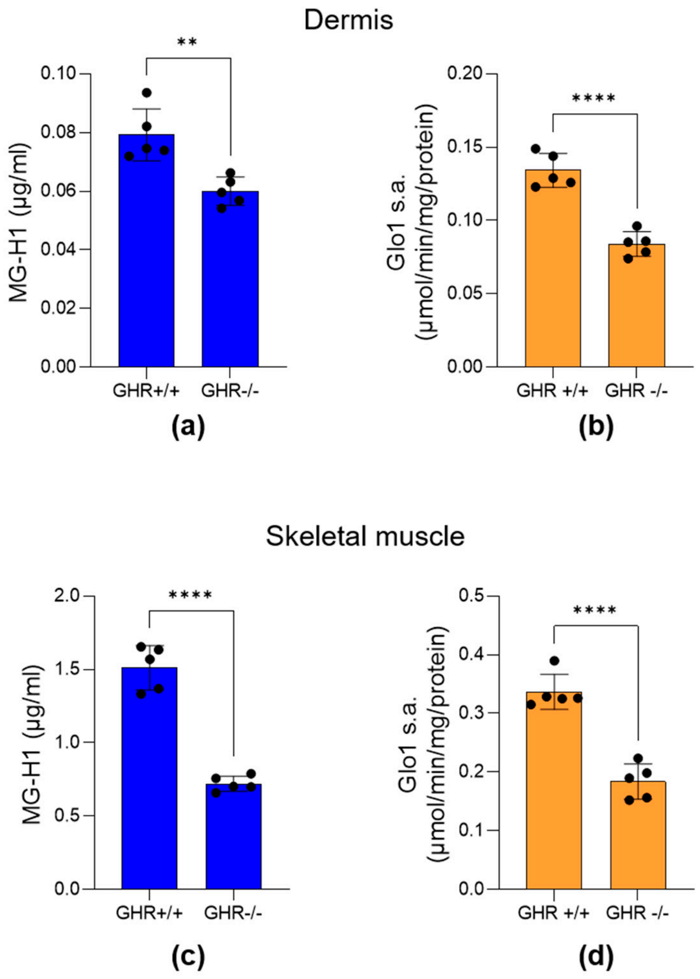

Figure S3. L-Carnosine reproduces the inhibitory effects of aminoguanidine on the IGF-1-induced pro-tumorigenic phenotype in LNCaP cells. LNCaP cells were pretreated with 10 mM L-carnosine for 48 h and subsequently stimulated with 150 ng/mL IGF-1 for 72 h in the continuous presence of L-carnosine. The effects of L-carnosine on **(a)** MG-H1 levels, **(b)** GLO1 enzymatic activity, **(c)** cell proliferation, **(d)** cell invasion, **(e)** RAGE mRNA expression, and **(f)** OPN mRNA expression were evaluated. L-Carnosine markedly attenuated the IGF-1-induced increase in MG-H1 accumulation, GLO1 activity, cell proliferation, invasion, RAGE expression, and OPN expression, reproducing the overall inhibitory effects observed with aminoguanidine. Data are presented as mean ± SD from three independent experiments. Statistical significance was determined by one-way ANOVA followed by Tukey's multiple comparison test. $^{**}p < 0.01$, $^{***}p < 0.001$, $^{****}p < 0.0001$

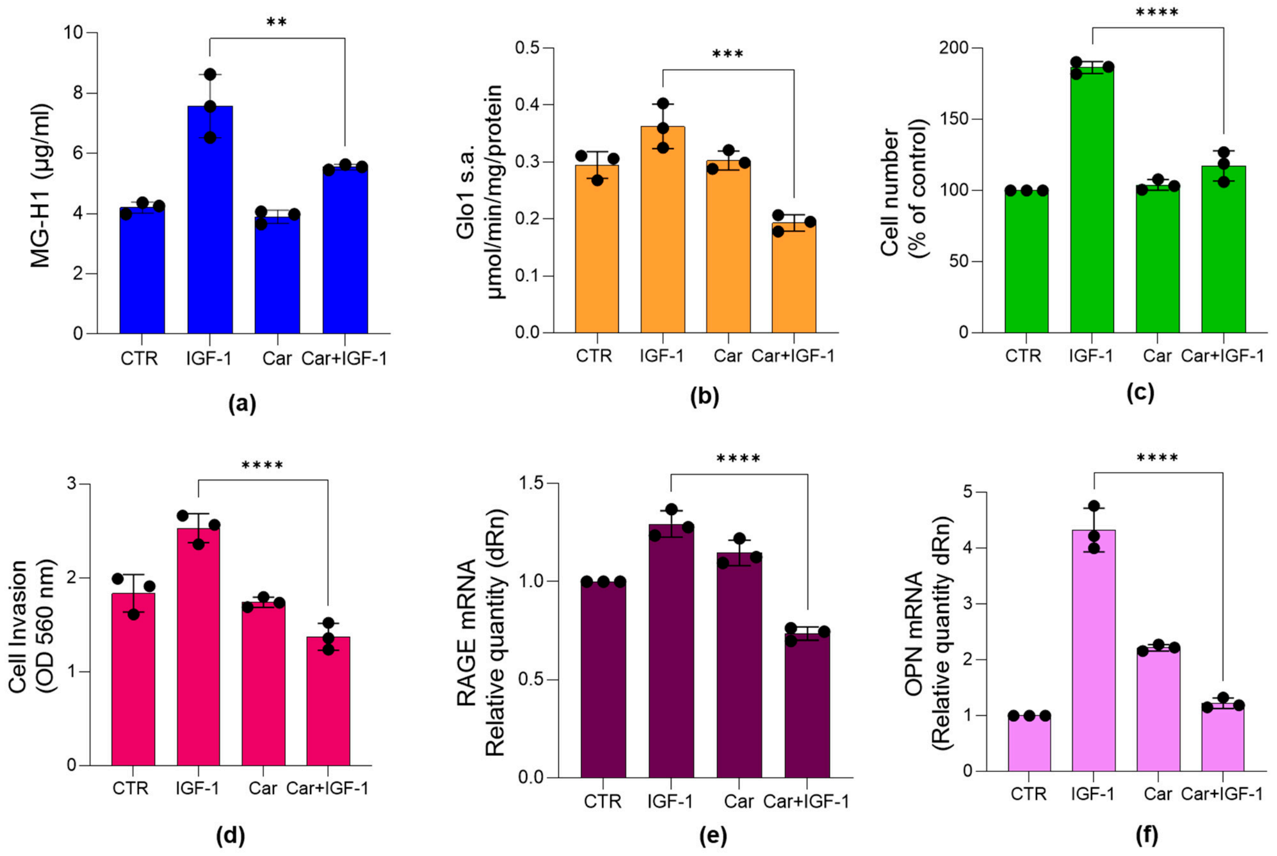

Figure S4. Uncropped Western blot images and original colony formation assay plates.

<u>Western blots</u>

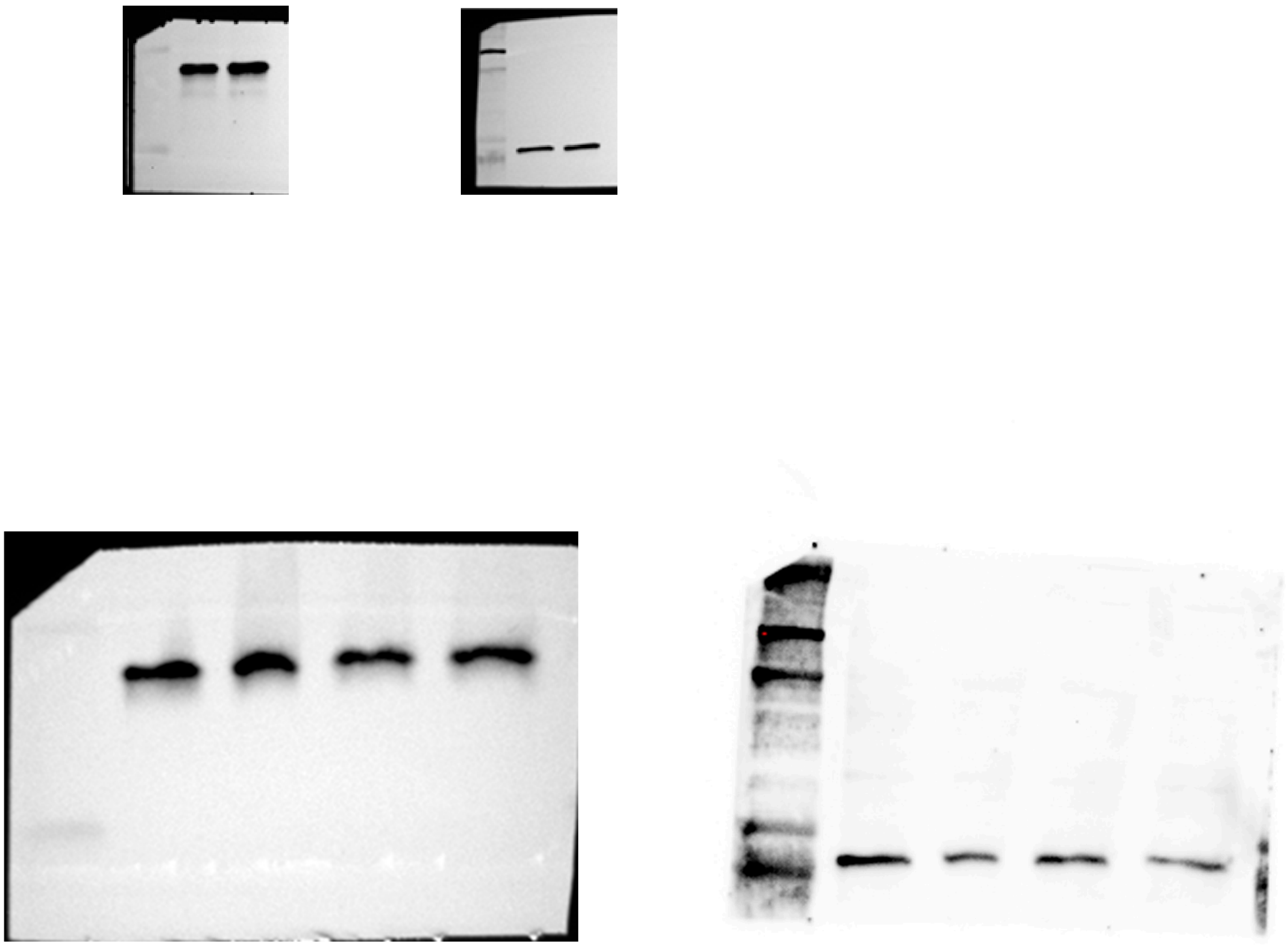

<u>Colony formation assay</u>. Original six-well plates from the same independent experiment are shown in their entirety for each treatment condition (Control, IGF-1, AG, and AG + IGF-1). Red circles identify the representative wells presented in Figures 4b and 8b of the main manuscript. For the Control and IGF-1 conditions, the representative images shown in Figures 4b and 8b correspond to different technical replicate wells from the same original plates. Quantitative analysis was performed using all replicates, as described in the Materials and Methods.

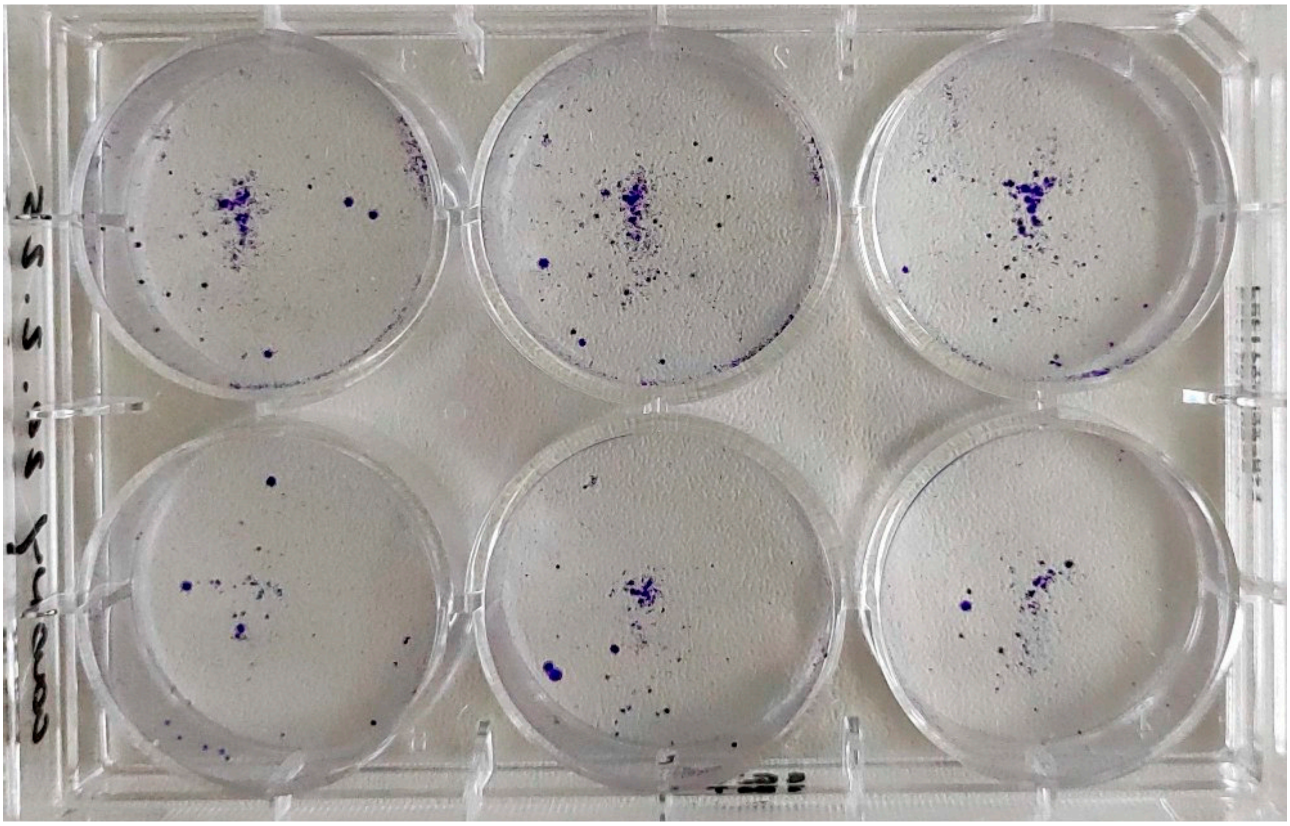

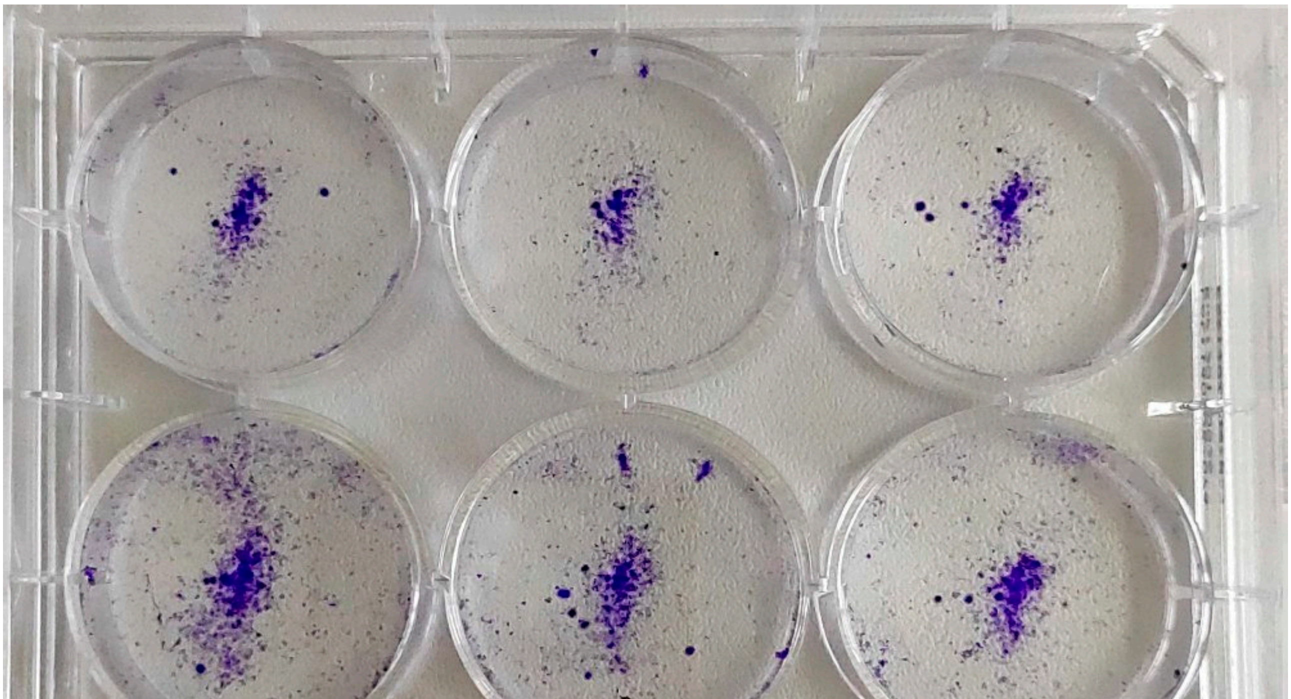

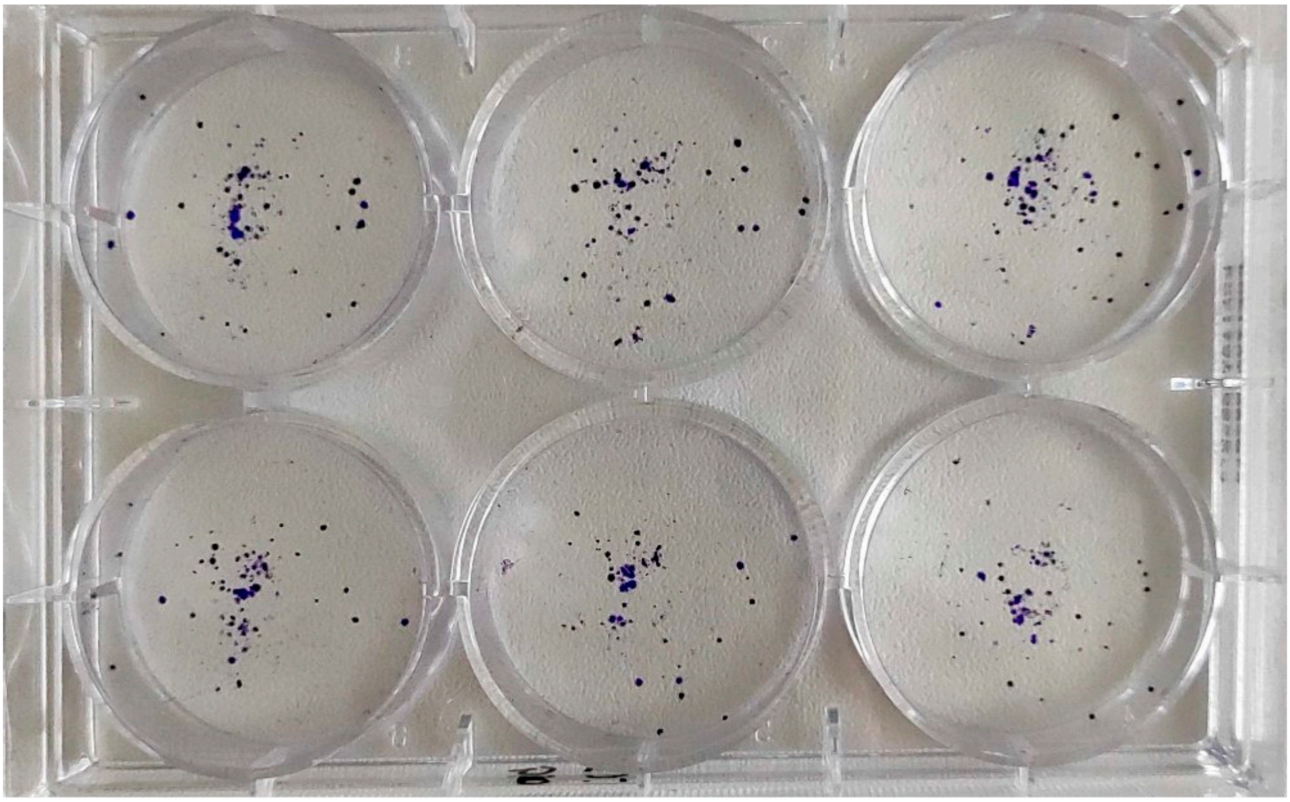

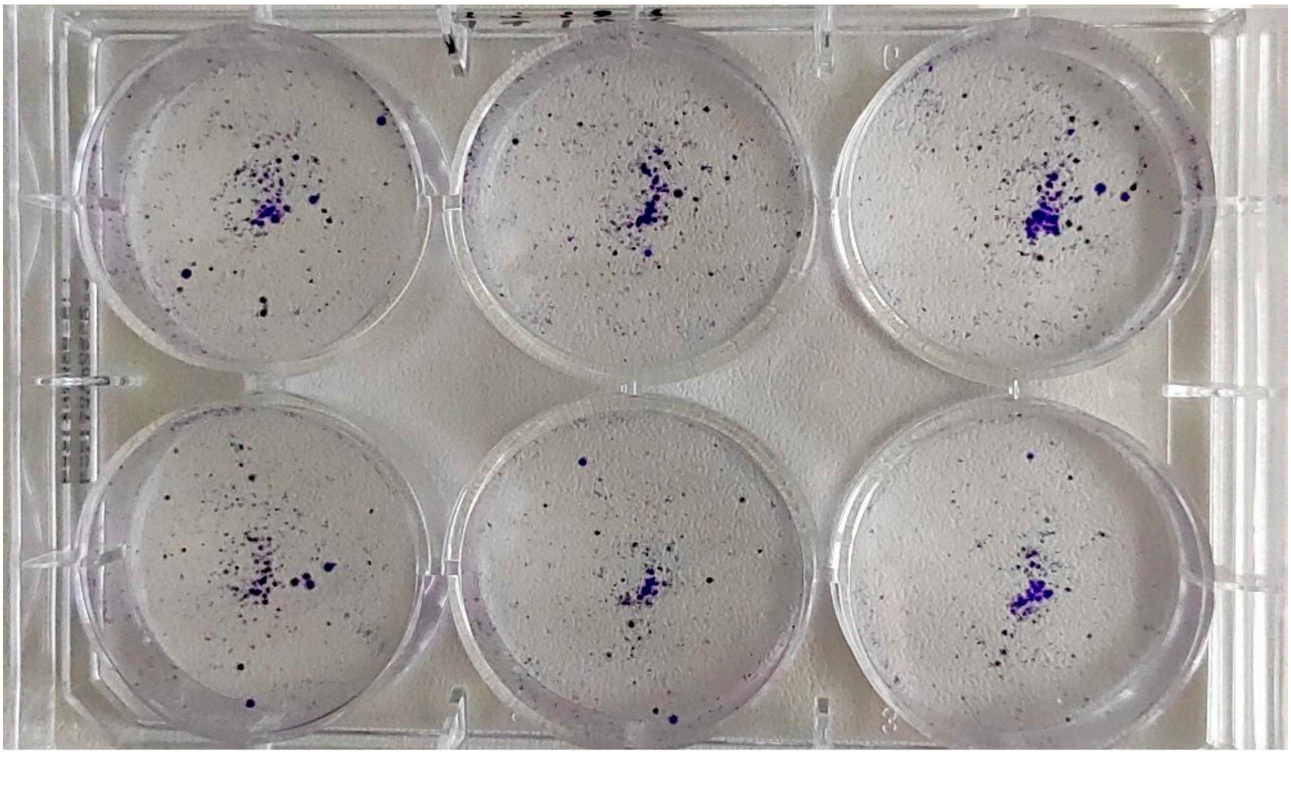

Supplement: Supplementary file 1 [file biology-15-01342-s001.zip › biology-4473423-supplementary.pdf]
